# Insulin‐like growth factor binding protein‐2 and glucose‐regulated protein 78 kDa: Potential biomarkers affect prognosis in *IDH* ‐wildtype glioblastoma patients

**DOI:** 10.1002/cam4.6071

**Published:** 2023-05-22

**Authors:** Abigail J. Harland, Claire M. Perks, Paul White, Kathreena M. Kurian, Hannah R. Barber

**Affiliations:** ^1^ Brain Tumour Research Centre, Bristol Medical School University of Bristol Bristol UK; ^2^ IGFs & Metabolic Endocrinology Group, Bristol Medical School, Translational Health Sciences University of Bristol, Southmead Hospital Bristol UK; ^3^ Faculty of Health Sciences University of the West England Bristol UK

**Keywords:** survival, cancer stem cells, prognosis, biomarkers

## Abstract

**Background:**

The overall survival of *IDH*‐wildtype glioblastoma patients is poor despite best available treatments. There is an urgent need for new biomarkers to inform more precise disease stratification. Previous studies have identified insulin‐like growth factor binding protein‐2 (IGFBP‐2) as a potential biomarker for glioblastoma diagnosis and therapeutic targeting. Other studies have indicated links between the insulin‐like growth factor (IGF) axis and tumorigenic functions of a molecular chaperone glucose related protein of 78 kDa (GRP78). We aimed to interrogate the oncogenic effects of IGFBP‐2 and GRP78 in our glioma stem cell (GSC) lines and clinical cohort.

**Methods:**

Immunoblotting, reverse transcription quantitative real‐time PCR were used to quantify protein and mRNA levels derived from GSCs and non‐malignant neural stem cells (NSCs). Microarray analysis was used to compare the differences in IGFBP‐2 (*IGFBP‐2*) and GRP78 (HSPA5) transcript expression between NSCs, GSCs and adult human cortex samples. Immunohistochemistry was used to quantify IGFBP‐2 and GRP78 expression in *IDH*‐wildtype glioblastoma tissue sections (*n* = 92) and clinical implications assessed using survival analysis. Finally, the relationship between IGFBP‐2 and GRP78 was further explored molecularly using coimmunoprecipitation.

**Results:**

Here, we demonstrate that *IGFBP‐2* and *HSPA5* mRNA is overexpressed in GSCs and NSCs in comparison to non‐malignant brain tissue. We also determined a relationship in which G144 and G26 GSCs expressed higher IGFBP‐2 protein and mRNA than GRP78, and this was reversed in mRNA isolated from adult human cortex samples. Clinical cohort analysis revealed that Glioblastomas with high IGFBP‐2 protein expression paired with low GRP78 protein expression were significantly associated with a much shorter survival time (Median = 4 months, *p* = 0.019) compared with 12‐14 months for any other combination of high/low protein expression.

**Conclusions:**

Inverse levels of IGFBP‐2 and GRP78 may be adverse clinical prognostic markers in *IDH*‐wildtype glioblastoma. Further interrogation of the mechanistic link between IGFBP‐2 and GRP78 may be important for rationalisation of their potential as biomarkers and therapeutic targets.

## 1 INTRODUCTION

Glioblastoma is the most prevalent and aggressive primary central nervous system (CNS) tumour with an incidence of 0.59–5 per 100,000 individuals.^2^ The median overall survival is approximately 15 months, with a 5‐year survival rate less than 5%.^3^ Survival time is largely dependent upon patient age, treatment modality and the molecular profile of the tumour.^4^, ^5^ Consequently, brain tumour patients face the highest mean average years of life lost compared with any other cancer.^6^ There is urgent need for new biomarkers to inform more precise disease stratification and to inform novel therapeutic options.^7^

Glioblastomas are heterogenous diseases, which are thought to comprise both differentiated tumour bulk cells and primitive glioma ‘stem‐like’ cells (GSCs), the latter of which represent a ‘moving target’ which may aid tumour recurrence and resistance to standard cytotoxic therapies.^8^, ^9^, ^10^, ^11^, ^12^ GSCs are therefore an important model for studying early cellular dysregulation as well as the adaptive mechanisms evoked under cellular stress. Molecular pathways integral to normal cell functioning have been implicated in driving poor glioblastoma survival and resistance, including the insulin‐like growth factor (IGF) axis and the chaperone glucose‐regulated protein of 78 kDa (GRP78).^13^, ^14^, ^15^

IGFBP‐2 is the second most abundant IGFBP in the human serum (after IGFBP‐3) and the most abundant in cerebral spinal fluid (CSF), here synthesised by choroid plexus epithelial cells and astroglia.^16^, ^17^ Established actions of IGFBP‐2 includes the binding of IGF‐I and IGF‐II with high affinity, modulating bioavailability and ligand‐IGF‐1R interactions.^18^ IGFBP‐2 expression is high during foetal and embryonic brain development and is significantly reduced after birth.^19^ Elevated levels are recapitulated in high grade glioma tissue biopsies and plasma samples in which poor clinical outcome potentiates harnessing IGFBP‐2 as outcome predictor and biomarker.^20^, ^21^, ^22^ IGFBP‐2 has also been delineated as part of a robust 9 gene signature associated with worse outcome for high‐grade glioma patients.^20^

The mechanism by which IGFBPs and molecular chaperones such as GRP78 influence glioma outcome is still unclear. However, experimental and clinical data have linked GRP78 and IGFBPs with carcinogenesis in the breast.^23^ GRP78 is a well‐established endoplasmic reticulum (ER) molecular chaperone and member five of the heat shock protein 70 (HSP70) superfamily; also known as binding immunoglobulin protein (BiP) or heat shock 70 kDa protein 5 (*HSPA5*).^24^ GRP78 is an important regulator of the unfolded protein response (UPR), by responding to changes in cell status via sequestration and release of signalling molecules vital for activation of anti‐apoptotic pathways.^24^ Its overexpression and cancer specific cell surface localisation, which have been described in glioblastoma, is believed to drive tumour growth, aggression and maintenance of the GSC mesenchymal phenotype.^13^, ^25^

In this study, we aimed to explore the expression of IGFBP‐2 and GRP78 protein and mRNA (*IGFBP‐2* and *HSPA5*) in our GSC and non‐malignant neural stem cell (NSC) lines and investigate potential protein biomarkers in our glioblastoma clinical biopsy cohorts with patient covariates and overall survival data.

## 2 METHODS

### 2.1 Formalin‐fixed paraffin‐embedded (FFPE) tissue samples

*IDH‐*wildtype glioblastoma tissue samples diagnosed between 15 March 2007 and 14 August 2014 were obtained under BRAIN UK ethical approval. Study Ref: 15/001.^1^ Brain UK REC Ref: 19/SC/0217 Renewal REC Ref: IRAS ID: 262890. Formalin‐fixed paraffin‐embedded (FFPE) tissue and clinical information was obtained for each patient. In some cases, clinical information was not available due to loss to clinical follow‐up or death.

### 2.2 Cell culture

Human Glioma stem cells (GSC) G144 and G26 and the U3 neural stem cell (NSC) were cultured as monolayers in serum‐free basal media. DMEM/F12 (Sigma, D8437) supplemented with N2 (Life Technologies, 17502048), B27 (Life Technologies, 17504‐044), mEGF (Preprotech, 315‐09, 10 ng/mL), human FGF (Preprotech, 100‐18C, 10 ng/mL) and laminin (Sigma, 11243217001, 1 μg/mL).^26^ All cells were cultured at 37°C with 5% CO_2_.

### 2.3 Protein and RNA extraction

Extraction of total protein from cells in lysis buffer (10 mM Tris hydrochloride (HCl) (Sigma)), 50 mM sodium chloride (NaCl, Sigma), 5 mM EDTA (Sigma), 1% (v/v) Triton X‐100 (Sigma), 15 mM tetrasodium pyrophosphate (Sigma), 50 mM sodium fluoride (Sigma), 100 μM sodium orthovanadate (Sigma), phosphatase (Sigma, P5726) and protease (Sigma, P8340) inhibitors (10 μL/1 mL lysis buffer) was quantified using a Pierce™ BCA (Bicinchoninic acid) kit (ThermoFisher Scientific, 23227), the iMark™ Microplate Reader (Bio‐Rad, UK) and accompanying Microplate Manager® Software.

Total RNA was extracted according to the manufacturers protocol for TRIzol (Ambion, 15596018). RNA was resuspended in 20 μL of DEPC‐treated water (Ambion, AM9906), heated at 58°C for 10 min before measuring the RNA concentration and purity using a nanophotometer (IMPLEN, P330).

### 2.4 Immunoblotting

30 μg of whole cell lysate, determined using a BCA assay were mixed with an equal volume of 2x Laemmli sample buffer concentrate and 10% 2‐mercaptoethanol (Sigma, S3041‐1VL) and heated at 95°C for 5 min in an AccuBlock™ digital dry bath (Labnet International, D1200). Protein separation was carried out by SDS‐PAGE and then transferred to a nitrocellulose membrane (Bio‐rad, 1620094). Non‐specific binding sites were blocked with 5% milk (Marvel) in tris‐buffered saline TWEEN®20 (TBS‐T) rocking for 60 min at room temperature. Membranes were incubated with specific primary antibodies diluted in fresh blocking solution overnight at 4°C: α‐tubulin (Millipore, 05‐829; 1:5000), IGFBP‐2 (Abcam, 109284; 1:1000), GRP78 (BD Transduction Laboratories™, 610979). Membranes were washed with TBS‐T and incubated with secondary antibodies, (Sigma, A0545 or A4416; 1:2000). Proteins were visualised exposing with the ChemiDoc MP Imaging System with Image Lab software (Bio‐Rad) once treated with Clarity Western ECL Substrate (Bio‐Rad, 1705061). Densiometric analysis of protein bands was carried out using Image J (NIH).

### 2.5 Co‐immunoprecipitation (Co‐IP)

T75 flasks at approximately 80% confluency were lysed with IP lysis buffer (20 mM Tris HCl pH 8 (Sigma), 137 mM NaCl (Sigma), 10% glycerol (Sigma), 1% Triton‐X‐100 (Sigma), 2 mM EDTA (ThermoFisher Scientific), phosphatase (Sigma, P5726) and protease (Sigma, P8340) inhibitors (10 μL/1 mL lysis buffer)). 500 μg of lysate, determined using a BCA assay was incubated with 2 μg of GRP78 primary antibody (610979) and 500 μg was incubated with 2 μg of IgG2a antibody (MAI‐10418) as an isotype negative control. The tubes were sealed with parafilm and incubated under rotary agitation overnight at 4°C. 60 μL of G Plus‐Agarose beads (Santa Cruz, sc‐2002) were equilibrated with IP lysis buffer. 30 μL of equilibrated beads were added to each sample and incubated under rotary agitation for 1 h at 4°C. After incubation, the beads were centrifuged at 2000 × g, 4°C and washed three times with IP lysis buffer. Proteins were eluted by adding 50 μL of 2 × Laemmli sample buffer concentrate and boiled at 95°C for 5 min. Samples were assessed using immunoblotting.

### 2.6 RT‐qPCR (reverse transcription‐quantitative PCR)

Reverse transcription from total RNA was carried out using the High‐Capacity RNA‐to‐cDNA Kit (ThermoFisher Scientific, 4387406). Resulting cDNA was loaded on 96 well, transparent, Low Profile, 100 μL plates (Sarstedt Ltd, 72.1981) with SYBR Green jumpstart Taq (‐20°C) ready mix (Sigma, S4438). The StepOne Plus Real‐Time PCR system (Applied Biosystems, 4376600) and StepOne software V2.3 was used for absolute quantification and melt curve analysis. The primer sequences used were as follows: *HSPA5* Forward: 5′‐GTGGCCACTAATGGAGATACTCATC and reverse: 5′‐GCCCGTTTGGCCTTTTCTAC. *IGFBP‐2* Forward: 5′‐GACAATGGCGATGACCACTCA and reverse: 5′‐GCTCCTTCATACCCGACTTGA. *GAPDH* Forward: 5′‐ACGGGAAGCTTGTCATCAAT and reverse: 5′‐TGGACTCCACGACGTACTCA.

### 2.7 Immunohistochemistry (IHC)

All immunohistochemistry was assessed by a trained consultant neuropathologist, expert in identifying glioma cells. To exclude expression in non‐neoplastic cells further, Haematoxylin and eosin‐stained sections were also compared to select sections with majority glioma cells.

IHC was performed on 92 *IDH*‐wildtype sections determined by immunohistochemistry with the R132HIDH1 antibody. This was followed up by IDH1/2 sequencing for non‐canonical IDH mutations in patients 55 years and younger according to the WHO 2021 guidelines. Sections were obtained from North Bristol NHS Trust as part of BRAIN UK.^1^ 3‐μm‐thick FFPE sections, collected on positively charged Apex Superior Adhesive Slides (Leica) with the primary monoclonal antibodies GRP78 (ab21685) and IGFBP‐2 (ab216957). All stages of the IHC were completed using a Ventana Benchmark ULTRA (Roche) fully automated IHC staining system. The staining system deparaffinised the slides and then applied CC2, pH 6 (GRP78) and CC1, pH 9 (IGFBP‐2) heat induced epitope retrieval. OptiView inhibitor (3% hydrogen peroxide) was used to quench endogenous peroxidases for 8 min at room temperature. The sections were incubated with the primary antibody at 1/200 for 32 min and then stained with the OptiView DAB IHC Detection Kit: HQ Universal Linker (secondary antibody cocktail containing HQ, a hapten covalently attached to goat antibodies) 8 min, horseradish peroxidase (HRP) multimer, 8 min, and 3,3′‐diaminobenzidine (DAB), 7 min. Any DAB staining was enhanced with OptiView copper sulphate (4 min) before counterstaining with Harris haematoxylin (Diapath) 1 min.

A double‐blind, semi‐quantitative system was used to quantify GRP78 and IGFBP‐2 immunohistochemical expression (Table 1). Overall scores were formed from addition of intensity and coverage scores, in which a score of ≥5 was used to indicate high GRP78 or IGFBP‐2 protein expression. Researchers blinded to the clinical data and independent of each other (K.M.K. and H.R.B.) assessed the staining. A consensus meeting was carried out to agree on expression scores in which a discrepant score was observed (a difference in combined score of ≥3 or a final difference in high or low outcome).

**TABLE 1:** The criteria used for IHC scoring.

Intensity	Score	Section percentage (%)	Score
No stain	0	No stain	0
Faint	1	1–25	1
Moderate	2	26–50	2
Strong	3	51–75	3
		76–100	4

### 2.8 Statistical analyses

Western blots and qPCR experiments were each repeated on three biological replicates in triplicate. Data are presented as mean ± SEM, except RT‐qPCR data (geometric mean ± 95% CI). Statistical tests were carried out using GraphPad (version 9.3.1 for Windows, GraphPad Software, www.graphpad.com), IBM SPSS Statistics 28.0.0.0 for Windows, and the Gliovis tool (http://gliovis.bioinfo.cnio.es/). [HG‐U133_Plus_2] Affymetrix Human Genome U133 Plus 2.0 Array data from Pollard et al. was sourced from the NIH Gene Expression Omnibus (series GSE15209), https://www.ncbi.nlm.nih.gov/geo/query/acc.cgi?acc=GSE15209. Data analyses were performed in collaboration with Miss Lily Andrews (Bristol Integrative Epidemiology Unit, University of Bristol). Data were downloaded and normalised in R studio (R version 4.0.3) using the caret package.^27^ One‐way ANOVA was used to compare the difference between three or more groups followed by the post hoc Tukey test or Dunnett test as appropriate. For comparing two groups, *t* tests were used. Kendall's Tau correlation coefficient was used to examine correlation of proteins in glioblastoma FFPE biopsy sections. Fisher's exact test was used to evaluate the significance of possible associations between GRP78 or IGFBP‐2 expression and clinicopathological covariates. OS was defined as the interval between patient diagnosis and death dates. The Log‐rank test was used to compare Kaplan–Meier survival curves. Univariate and multivariate Cox proportional hazards regression analysis were used to calculate hazard ratios and associated 95% confidence intervals. All statistical tests were two‐tailed. Differences with *p* < 0.05 were considered statistically significant.

### 2.9 IGFBP‐2 is overexpressed in stem cells and upregulated in GSCs

**FIGURE 1:**
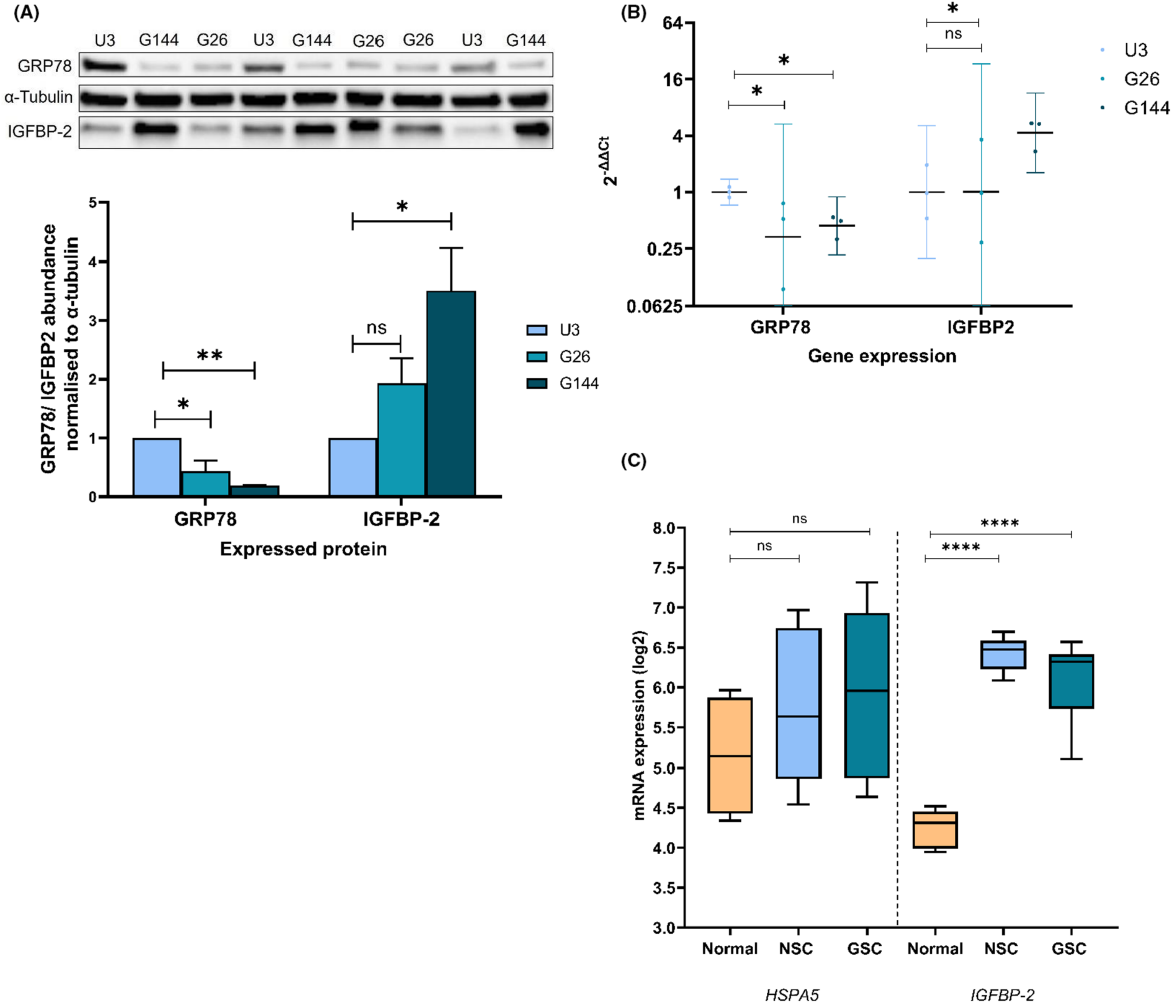
Protein and gene expression of GRP78 and IGFBP‐2 in G26, G144 GSC cells, U3 control neural stem cells (NSCs) and normal brain (def). (A) Immunoblot analysis of GRP78 and IGFBP‐2 protein expression in three independent passages of the control U3 NSC line and two GSC lines, *n* = 3. Densiometric analysis below the representative immunoblot was used to quantify protein expression levels for three technical repeats. After normalisation of protein integrated area density values relative to the loading control α‐tubulin values, ratiometric analysis was used to examine the GSC expression values relative to grouped U3 control protein homogenate expression. Data are presented as mean ± SEM, *n* = 3 biological repeats, all samples were analysed in triplicate. Additional technical repeats are shown in supporting information. (B) *HSPA5* and *IGFBP‐2* mRNA expression for three independent passages of the control U3 NSC line and two GSC lines. Examined by RT‐qPCR and normalised to the reference gene *GAPDH*. 2^−ΔΔCt^ was calculated to represent fold change. Data are presented as geomean and 95% confidence interval on a logarithmic scale (base 2), *n* = 3 biological repeats, all samples were analysed in triplicate. (C) Box plots depicting *IGFBP‐2* and *HSPA5* expression from human foetal NSC lines, GSCs and normal brain. ****p < 0.0001; ***p = 0.0001–0.001; **p = 0.001–0.01; *p = 0.001 to 0.05; ns, not significant *p* ≥ 0.05.

To assess the relative basal expression of GRP78 and IGFBP‐2, 3 independent passages of G26 and G144 GSCs and non‐malignant U3 NSCs were analysed using western blotting and qPCR. The fold‐change expression for GRP78 and IGFBP‐2 in GSCs was then calculated with respect to the U3 NSCs. Staining for GRP78 was the strongest in the non‐tumorigenic U3 cells with significantly lower expression in G26 and G144 cells (*p* = 0.023 and *p* = 0.004 respectively, Figure 1A). IGFBP‐2 staining was strongest in the G144 cells and significantly increased compared to U3 cells (*p* = 0.019). In addition, there was significantly lower *HSPA5* mRNA expression isolated from G26 and G144 cells (*p* = 0.038 and *p* = 0.036 respectively, Figure 1B) compared to U3 when normalised to *GAPDH* expression. The expression of *IGFBP‐2* was significantly higher in G144 than U3 cells (*p* = 0.048).

Expression profiling data were then analysed for differences in *IGFBP‐2* and *HSPA5* transcript expression between NSCs, GSCs and adult human cortex samples (Gene Expression Omnibus (GEO) accession number GSE15209). *IGFBP‐2* transcript expression was significantly increased in GSCs and NSCs in comparison to normal brain (*p* < 0.0001, Figure 1C). Differences in *HSPA5* transcript expression did not reach significance. Pooled expression values in this data revealed a pattern suggesting that stem cells express higher levels of *IGFBP‐2* than *HSPA5* and this is reversed for differentiated brain samples. Despite the significant differences in GRP78 and IGFBP‐2 expression observed in this study between the U3 NSCs, G26 and G144 GSCs, microarray data suggest that both IGFBP‐2 and GRP78 appear to be expressed at higher levels in GSCs and NSCs. Pooled expression values reveal a pattern, suggesting that stem cells express higher levels of IGFBP‐2 than HSPA5 and this is reversed for differentiated brain samples.

### 2.10 GRP78 and IGFBP‐2 protein expression in glioblastoma patient cohort biopsy tissue

**FIGURE 2:**
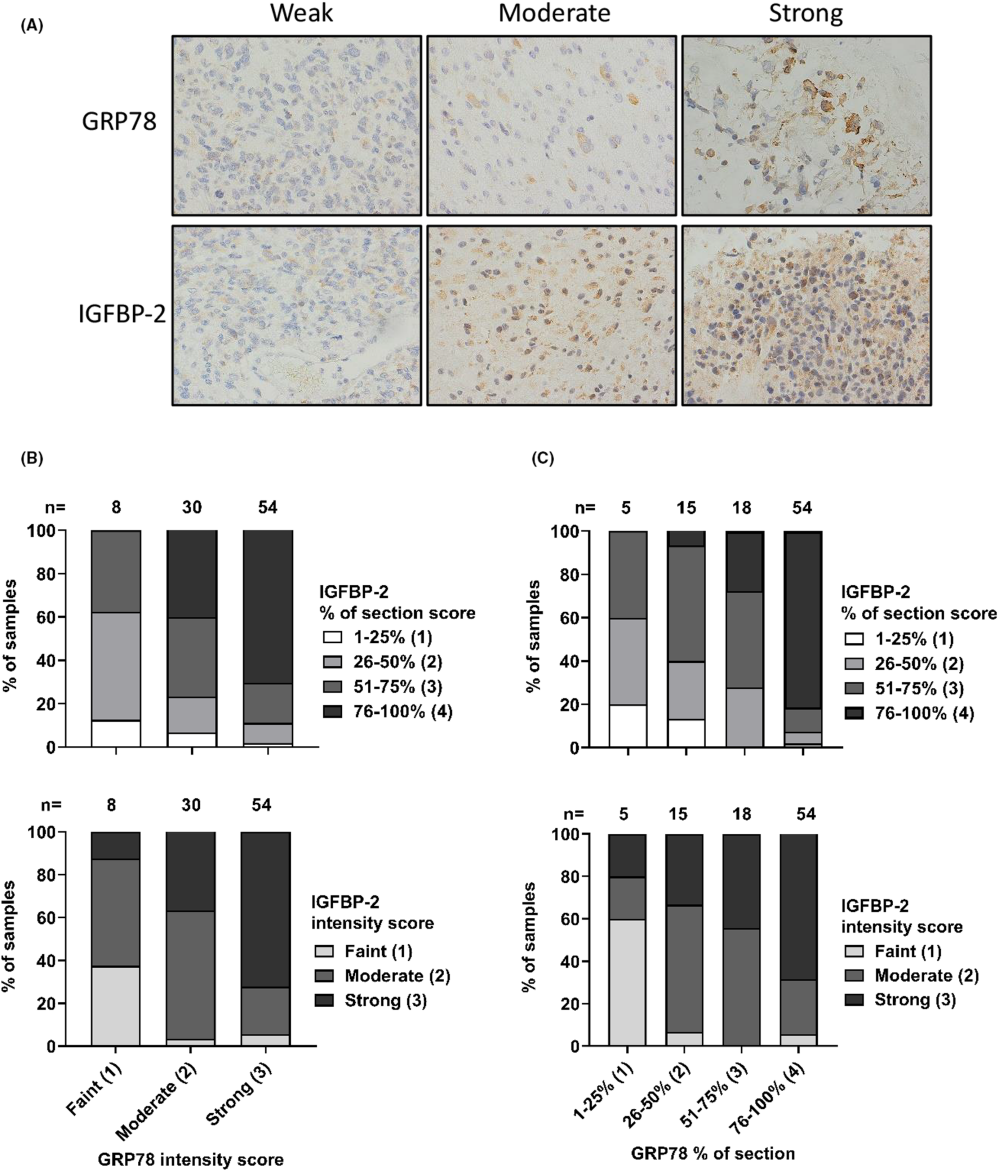
IGFBP‐2 and GRP78 immunohistochemistry (IHC) staining in 92 *IDH‐*wildtype glioblastoma biopsy sections. Expression levels of IGFBP‐2 and GRP78 were detected using IHC from a clinical cohort including 92 *IDH1*‐wildtype glioblastoma biopsy sections. (A) IHC representative images showing GRP78 and IGFBP‐2 weak, moderate and strong staining. Images taken at × 40 magnification. (B) GRP78 intensity score associated with IGFBP‐2 intensity score or section coverage. The stacked bar charts depict increasing IGFBP‐2 section coverage and staining intensity with respect to GRP78 staining intensity. (C) Section coverage percentage of GRP78 associated with section coverage percentage or intensity score of IGFBP‐2. The stacked bar chart depicts increasing IGFBP‐2 section coverage and staining intensity with respect to GRP78 section coverage.

IHC was performed on 92 glioblastoma (*IDH*‐wildtype) FFPE biopsy sections to assess levels and association between GRP78 and IGFBP‐2. The double‐blind, semi‐quantitative criteria used to score GRP78 and IGFBP‐2 staining is detailed in Table 1. Example IHC images indicating weak, moderate and strong GRP78 and IGFBP‐2 staining are shown in Figure 2A. We observed that with increasing GRP78 intensity score, both the IGFBP‐2 intensity and section coverage score increased. Moreover, sections which had a higher score for GRP78 staining coverage also had a larger IGFBP‐2 coverage score (Figure 2B,C).

### 2.11 Patient cohort analysis of GRP78 and IGFBP‐2 IHC scores, clinicopathological features and OS

Further analysis of the *IDH‐*wildtype glioblastoma cohort (*n* = 92) was carried out. Diagnosis occurred between the 15 March 2007 and 14 August 2014. Four patients were lost to follow‐up, censoring occurred on the 1 May 2016. The clinicopathological features of this cohort are summarised in Table 2. Using double‐blind semi‐quantitative criteria, GRP78 IHC scores were interpreted as ‘high’ in 74 (80.4%) and ‘low’ in 18 glioblastoma samples (19.6%). IGFBP‐2 IHC scores were interpreted as ‘high’ in 75 (81.5%) and ‘low’ in 17 glioblastoma samples (18.5%). The associations between GRP78 or IGFBP‐2 and the clinicopathological features of this cohort were analysed (Table 2), where the *p* value indicates the significance of the association. No significant associations were reported between GRP78 or IGFBP‐2 IHC scores and patient sex, age, KPS, tumour location or *MGMT* promoter methylation status. (Table 2, *p* > 0.05 for all covariates).

**TABLE 2:** Associations between GRP78 or IGFBP‐2 IHC protein expression and clinicopathological features of *IDH1‐*wildtype glioblastoma (*n* = 92). The Fisher's exact test, two‐tailed *p* value was used to evaluate significance.

Clinicopathological variables	GRP78				IGFBP‐2		
	Patients	High	Low	*p* value	High	Low	*p* value
Sex				0.181			0.401
Male	59	50	9		50	9	
Female	33	24	9		25	8	
Age (years)				0.846			0.670
28–55	33	27	6		27	6	
56–65	29	24	5		25	4	
66–83	30	23	7		23	7	
KPS^a^				0.721			0.722
≥80	60	47	13		48	12	
<80	15	13	2		13	2	
Tumour location^b^				0.542			0.336
Frontal	21	18	3		19	2	
Non‐frontal/diffuse	63	49	14		49	14	
MGMT status^c^				1.0			0.591
Methylated	45	36	9		38	7	
Unmethylated	45	36	9		35	10	

^a^ 17 missing data points.

^b^ 8 missing data points.

^c^ 2 missing data points.

Abbreviations: GRP78, 78‐kDa Glucose‐regulated protein; IGFBP‐2, Insulin Like Growth Factor Binding Protein 2; KPS, Karnofsky performance score; *MGMT*, O^6^‐methylguanine‐DNA methyltransferase promoter.

**FIGURE 3:**
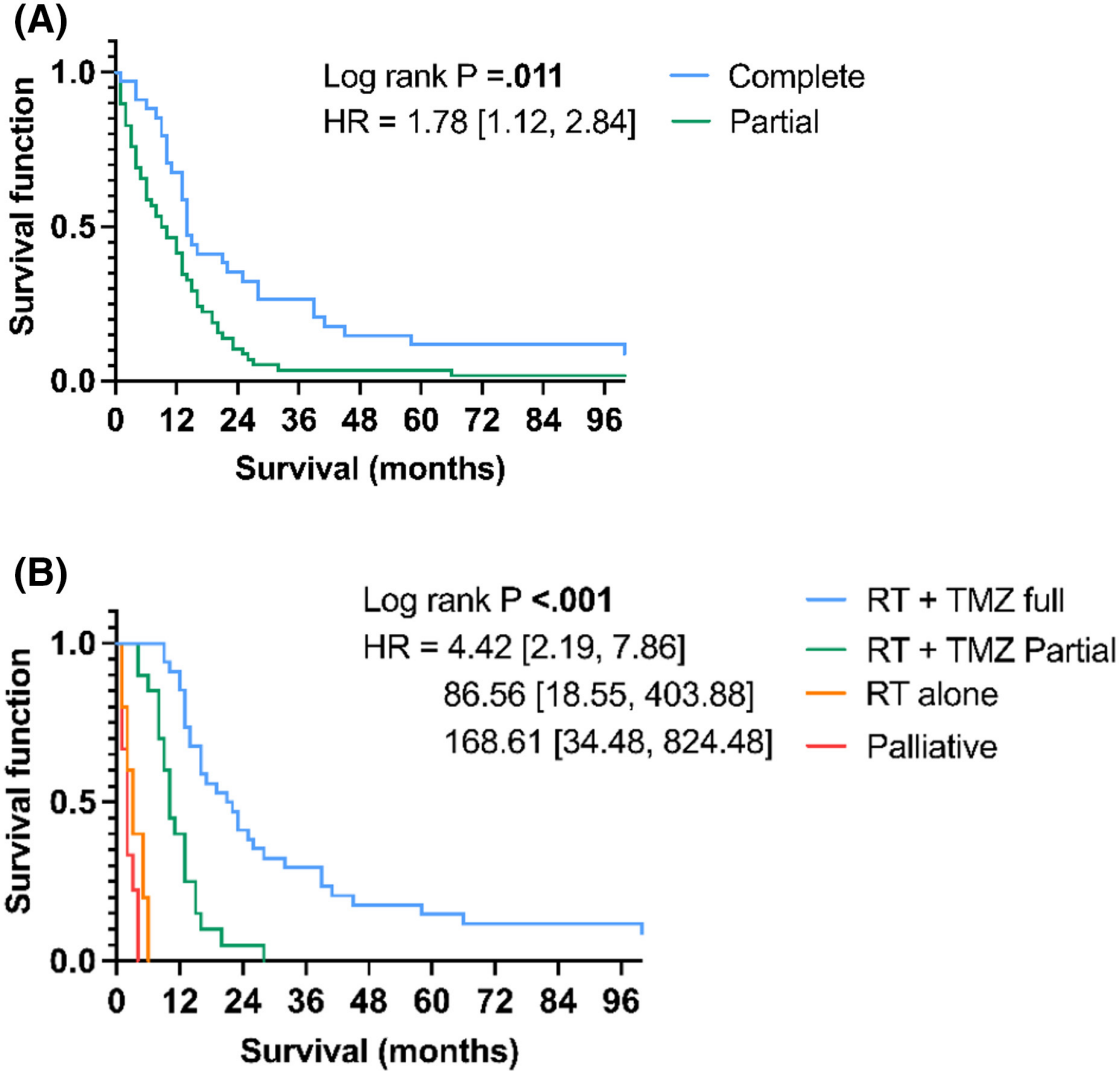
Kaplan–Meier analysis showing associations between patient overall survival (OS) and clinical covariates of 92 primary *IDH‐*wildtype glioblastoma patients. All Log rank *p* < 0.05 (A) Patient extent of tumour resection analysis. (B) Patient treatment analysis. CI, confidence interval; CNS, central nervous system; HR, hazard ratio; KPS, karnofsky performance scale; MGMT, O^6^‐methylguanine‐DNA methyltransferase promoter; RT, Radiotherapy; TMZ, temozolomide.

Patient overall survival (OS) times were then analysed for associations with clinical covariates, including extent of surgical resection and adjuvant treatment modality (Table 3). Patient OS was calculated in months from diagnosis. In the glioblastoma (*IDH‐*wildtype) clinical cohort (*n* = 92), the median overall survival (OS) for all patients was 12 months (range: 1–100 months), from the date of diagnosis. Univariate Cox's proportional hazards analysis of clinical covariates demonstrated no significant correlation between patient sex, age, KPS, tumour location, *MGMT* promoter methylation, GRP78, IGFBP‐2 glioblastoma tissue abundance and OS (*p* > 0.05, Table 3). However, patient extent of surgical resection, and treatment modality were significantly correlated with the OS of glioblastoma patients (*p* = 0.011 and *p* < 0.001 respectively, Table 3, Figure 3).

**TABLE 3:** Univariate Kaplan Meier and Cox proportional hazards analysis of associations between OS, clinical covariates and GRP78/ IGFBP‐2 IHC protein expression of *IDH1‐*wildtype glioblastoma (*n* = 92). Log rank *p* and HR values were used to compare groups.

Clinicopathological variable	*n* (events)	Median OS (months) [95% CI]	Univariate	
			*p* value	HR [95% CI]
Sex			0.340	
Male	59 (55)	13.00 [11.58, 14.42]		1
Female	33 (33)	9.00 [5.62, 12.37]		0.815 [0.53, 1.26]
Age (years)			0.128	
28–55	33 (29)	14.00 [11.36, 16.64]		1
56–65	29 (29)	9.00 [3.73, 14.27]		1.68 [0.99, 2.85]
66–83	30 (30)	12.00 [7.97,16.03]		1.25 [0.74, 2.10]
KPS^a^			0.333	
≤ 80	29 (29)	11.00 [5.73, 16.27]		1
81–90	24 (22)	13.00 [11.25, 14.76]		1.08 [0.61, 1.93]
91–100	22 (20)	15.00 [12.08, 17.92]		0.71 [0.40, 1.26]
Tumour location^b^			0.491	
Frontal	21 (20)	10.00 [7.09, 12.91]		1
Non‐frontal	62 (60)	13.00 [11.71, 14.26]		0.94 [0.56, 1.56]
Diffuse	1(1)	7.00 [–]		2.87 [0.37, 22.04]
Resection^c^			**0.011**	
Complete	34 (31)	14.00 [11.69, 16.31]		1
Partial	52 (51)	9.00 [4.00, 14.00]		1.78 [1.12, 2.84]
MGMT status^d^			0.344	
Methylated	45 (42)	10.00 [4.71, 15.29]		1
Unmethylated	45 (44)	12.00 [9.56, 14.44]		1.23 [0.79, 1.90]
Treatment^e^			**<0.001**	
RT + TMZ full	34 (31)	20.00 [13.46, 26.54]		1
RT + TMZ partial	20 (20)	10.00 [8.57, 11.43]		4.42 [2.19, 7.86]
RT alone	5 (5)	3.00 [0.85, 5.17]		86.56 [18.55, 403.88]
Palliative	9 (9)	2.00 [1.08, 2.92]		168.61 [34.48, 824.48]
GRP78			0.509	
High	74 (71)	13.00 [10.27, 15.73]		1
Low	18 (17)	10.00 [1.93, 18.07]		0.84 [0.49, 1.43]
IGFBP‐2			0.395	
High	75 (71)	10.00 [7.42, 12.58]		1
Low	17 (17)	14.00 [12.01, 15.99]		1.25 [0.73, 2.13]

^a^ 17 missing data points.

^b^ 8 missing data points.

^c^ 6 missing data points.

^d^ 2 missing data points.

^e^ 24 missing data points.

*Note*: *RT + TMZ full*: 60Gy total given over 5 days/ week for 6 weeks with concurrent daily temozolomide (75 mg/m^2^/day, 7 days/week) and 6 cycles of adjuvant TMZ (150–200 mg/m^2^/day for 5 days during each 28‐day cycle). *RT + TMZ Partial*: 60Gy total given over 5 days/ week for 6 weeks with concurrent daily temozolomide (75 mg/m^2^/day, 7 days/week) without completing 6 cycles of adjuvant TMZ; or 60Gy total given over 5 days/ week for 6 weeks without concurrent temozolomide but completing 6 cycles of adjuvant TMZ (150–200 mg/m^2^/day for 5 days during each 28‐day cycle. *n*, number of people in each group in Table 3. Events, indicates the number of people that died in each group in Table 3. Bold indicates statistical significance (*p* < 0.05).

Abbreviations: CI confidence interval; GRP78, 78‐kDa glucose‐regulated protein; HR, hazard ratio; IDH1, Isocitrate dehydrogenase 1; IGFBP‐2, insulin like growth factor binding protein 2; IHC, immunohistochemistry; KPS, karnofsky performance score; MGMT, O^6^‐methylguanine‐DNA methyltransferase promoter; OS, overall survival; RT, radiotherapy; TMZ, temozolomide.

Patients aged 28–55 years (median survival = 14.00 months) survived significantly longer than for patients aged between 56 and 65 years (median survival = 9.00 months HR: 1.68 [95% CI, 0.99, 2.85]) (*p* = 0.042), but not significantly longer than patients aged between 66 and 83 years (median survival = 12.00 months, HR: 1.25 [95% CI, 0.74, 2.10]), (*p* < 0.001, Table 3). The survival of patients that underwent complete resection (median survival = 14.00 months) was significantly longer than that of patients that underwent partial resection (median survival = 9.00 months, HR: 1.78 [95% CI, 1.12, 2.84]), (*p* = 0.011). In addition, patients that underwent the full Stupp protocol (60Gy total given over 5 days/week for 6 weeks with concurrent daily temozolomide (75 mg/m^2^/day, 7 days/week) and 6 cycles of adjuvant temozolomide (150–200 mg/m^2^/day for 5 days during each 28‐day cycle) survived significantly longer (median survival = 20.00 months) than those that received partial Stupp treatment (60Gy total given over 5 days/week for 6 weeks with concurrent daily temozolomide (75 mg/m^2^/day, 7 days/week) but without completing 6 cycles of adjuvant temozolomide); or 60Gy in 30 fractions without concurrent temozolomide but completing 6 cycles of adjuvant temozolomide) (median survival = 10.00 months, HR: 4.42 [95% CI, 2.19, 7.86]), radiotherapy treatment alone (median survival = 3.00 months, HR: 86.56 [95% CI, 18.55, 403.88]) or palliative care (median survival = 2.00 months, HR: 168.61 [95% CI, 34.48, 824.48]) (*p* < 0.001, Table 3). The significant associations between patient resection status, patient treatment and overall survival are displayed as Kaplan–Meier curves (Figure 3).

### 2.12 Patient survival differences with GRP78 and IGFBP‐2 combinations

**FIGURE 4:**
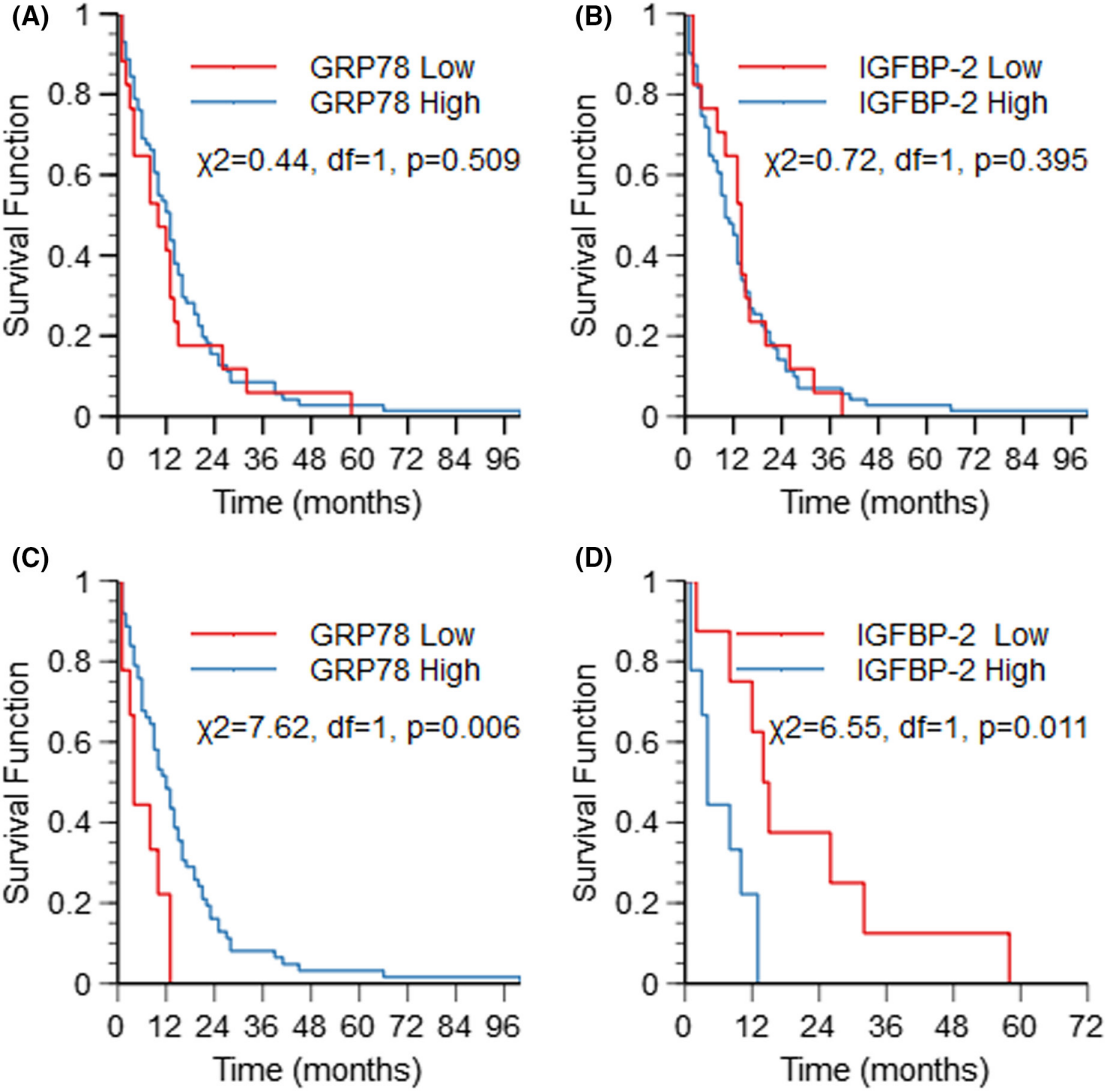
Kaplan–Meier analysis showing associations between patient overall survival (OS) and combinations of GRP78high/low and IGFBP‐2high/low expression in 92 primary *IDH‐*wildtype glioblastoma patient tumours. (A) GRP78 is not related to survival (*p* = 0.509). (B) IGFBP‐2 is not related to survival (*p* = 0.395). (C) When IGFBP‐2 is high there is a significant survival difference between high and low GRP78 (*p* = 0.006). (D) When GRP78 is Low, survival is dependent on whether IGFBP‐2 is High or Low (*p* = 0.011).

In our adult *IDH‐*wildtype glioblastoma patient cohort (*n* = 92), no significant associations were found between GRP78 or IGFBP‐2 and patient OS. We next analysed the relationship between GRP78 and IGFBP‐2 using the chi‐squared test. In this cohort, GRP78 and IGFBP‐2 IHC scores were shown to be significantly associated (*p* = 0.002) and correlated (gamma =0.705, *p* = 0.016). Moreover, combining GRP78 and IGFBP‐2 into four variables based on high and low IHC scores (IGFBP‐2_low_; GRP78_high_, IGFBP‐2_low_; GRP78_low_, IGFBP‐2_high_; GRP78_high_ and IGFBP‐2_high_; GRP78_low_), revealed that for patients with high IGFBP‐2 and low GRP78 tumour abundances there was a significantly shorter median survival time when compared with to the other groups (Table 4, IGFBP‐2_high_; GRP78_low_ median OS = 4 months, *p* = 0.019). Kaplan–Meier curves show the four different combinations and patient outcome (Figure 4). Further analysis demonstrated that when GRP78 is low, survival is dependent on whether IGFBP‐2 is high or low (*p* = 0.011, Figure 4D), and when IGFBP‐2 is high there is a significant survival difference between high and low GRP78 (*p* = 0.006, Figure 4C).

**TABLE 4:** Univariate Kaplan–Meier and Cox proportional hazards of associations between OS, and combinations of IGFBP‐2/GRP78 IHC protein expression of *IDH1‐*wildtype glioblastoma (*n* = 92). Log rank *p* and HR values were used to compare groups.

Variable	*n*	Median OS (months)	Standard error	95% CI	
				Lower bound	Upper bound
IGFBP‐2_low_; GRP78_high_	9	13.0	2.24	8.62	17.38
IGFBP‐2_low_; GRP78_low_	8	14.0	2.12	9.84	18.16
IGFBP‐2_high_; GRP78_high_	62	12.0	1.75	8.57	15.43
IGFBP‐2_high_; GRP78_low_	9	**4.0**	**0.75**	**2.54**	**5.46**

The nine patients with high IGFBP‐2 and low GRP78 tumour expression were further assessed for similarities based on patient characteristics and clinical covariates (Table 5). Four patients were positive for *MGMT* promoter methylation and five were negative. Two patients had tumour biopsies, three underwent partial tumour resection, three underwent complete resection and one patient's resection status was unavailable. The locations of the tumours also showed differences, with two patients presenting with right temporal tumours, one with right parietal, one with right frontal, one with left frontal, one with left occipital, one with corpus callosum, one with bilateral parietal and one location was unavailable. The survival times of the three females in the group ranged from 1 to 4 months, whilst the 6 males ranged from 1 to 13 months.

**TABLE 5:** Clinicopathological variables and covariates for 9 patients with a high IGFBP‐2/low GRP78 IHC protein expression in a cohort of 92 patients with *IDH1‐*wildtype glioblastoma.

ID	Sex	Age (years)	KPS	Tumour location	Resection	MGMT status	Treatment	OS (months)
2012/0940	Male	45	70	Left frontal	Partial	M	RT + TMZ partial	10
2013/0636	Female	70	80	Right parietal	Complete	M	NA	4
2012/0884	Male	48	90	Right frontal	Complete	U	RT + TMZ partial	8
2008/054	Male	69	90	Right temporal	Partial	U	RT + TMZ partial	13
2008/0057	Male	57	90	Right temporal	Partial	U	RT + TMZ full	13
2012/0311	Female	71	100	Left occipital	Complete	M	RT + TMZ partial	4
12/868	Male	83	100	Corpus callosum	Biopsy	U	RT + TMZ partial	3
2013/0845	Female	59	NA	Bilateral parietal	Biopsy	M	NA	1
2013/0306	Male	55	NA	NA	NA	U	NA	1

*Note*: *RT + TMZ full*: 60Gy total given over 5 days/ week for 6 weeks with concurrent daily temozolomide (75 mg/m^2^/day, 7 days/week) and 6 cycles of adjuvant TMZ (150–200 mg/m^2^/day for 5 days during each 28‐day cycle). *RT + TMZ Partial*: 60Gy total given over 5 days/week for 6 weeks with concurrent daily temozolomide (75 mg/m^2^/day, 7 days/week) without completing 6 cycles of adjuvant TMZ; or 60Gy total given over 5 days/ week for 6 weeks without concurrent temozolomide but completing 6 cycles of adjuvant TMZ (150–200 mg/m^2^/day for 5 days during each 28‐day cycle.

Abbreviations: ID, patient identifier; KPS, karnofsky performance score; M, methylated; MGMT, O^6^‐methylguanine‐DNA methyltransferase promoter; NA, not available; OS, Overall survival; RT, radiotherapy; TMZ, temozolomide; U, unmethylated.

**FIGURE 5:**
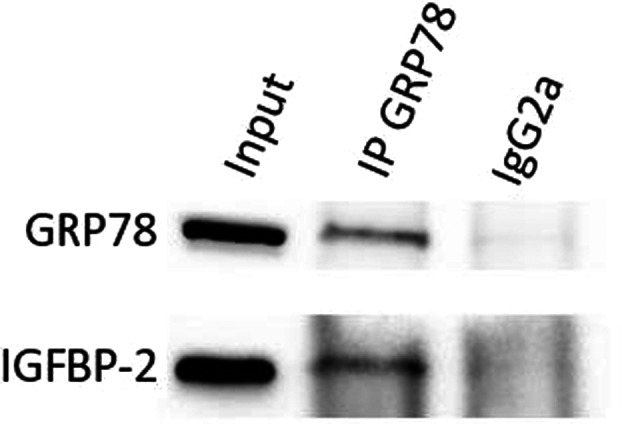
Immunoblot showing a co‐IP assay carried out on lysates extracted from G26 GSCs. IGFBP‐2 was pulled down using a GRP78 antibody. The IgG2a antibody was used as an isotype control. Immunoblots from two additional co‐IP assays are shown in supporting information.

### 2.13 The association between GRP78 and IGFBP‐2 in GSCs

To explore a mechanistic link between GRP78 and IGFBP‐2, we carried out co‐immunoprecipitation experiments. A GRP78 antibody was used to immunoprecipitate G26 cell lysates. The western blot shows the interaction between GRP78 and IGFBP‐2 (Figure 5). A IgG2a isotype control antibody was used as a comparison.

## 3 DISCUSSION

IGFBP‐2 appears to play key roles in foetal development and cognitive development.^28^, ^29^, ^30^, ^31^ Studies have shown that the IGFBP‐2 genes promoter also contains binding sites for transcription factors involved in the maintenance of a stem cell state.^14^ In our in vitro analysis of protein levels, we observed elevated IGFBP‐2 expression in G26 and G144 GSC lines compared to U3 NSC lines, raising the possibility that the developmental pathways mediated by IGFBP‐2 were aberrantly activated in these cells. GRP78 is critical for cell fate, controlling the survival/apoptotic potential of the cell in response to stress. GRP78 overexpression has been reported in glioblastoma, correlating with aggressive and radioresistant phenotypes.^13^, ^32^ Our cell line work and microarray analysis indicated that IGFBP‐2 and GRP78 appear to be expressed at higher levels in GSCs and NSCs. Stem cells also showed higher expression levels of IGFBP‐2 than HSPA5 and this was reversed for differentiated brain samples. In our individual cell lines, GRP78 protein and mRNA (HSPA5) expression was lower than IGFBP‐2 in G26 and G144 GSCs. However, the analysis of more cells including a range of subtypes is required to determine whether this expression pattern could define a subset of GSCs. A similar relationship has been reported with IGFBP‐3 and GRP78, where the combination of low GRP78 and high IGFBP‐3 was clinically significant, indicating poorer overall survival of breast cancer patients.^23^ Finally, we carried out co‐IP experiments in lysates collected from the G26 cell line to confirm the mechanistic association between GRP78 and IGFBP‐2.

We quantified GRP78 and IGFBP‐2 protein expression for 92 *IDH*‐wildtype glioblastoma patient tumour samples. Survival analysis between the clinical covariates of these patients demonstrated that patient extent of surgical resection, and treatment modality were significantly correlated with the OS of glioblastoma patients (*p* = 0.011 and *p* < 0.001 respectively). However, MGMT promoter methylation status did not reach significance with respect to overall survival. MGMT is an important predictive biomarker and indication of a patient's sensitivity to alkylating chemotherapy. In our small cohort study, 26.7% of patients with known *MGMT* promoter methylation status (*n* = 90) had no information on treatment received. Of those patients in which treatment modality was available, more patients without *MGMT* promoter methylation received some kind of alkylating therapy (whether full or partial), and more patients with *MGMT* promoter methylation received palliative care. Further survival analysis using the clinical tumour biopsy samples demonstrated that neither IGFBP‐2 nor GRP78 expression were significantly associated with survival alone (IGFBP‐2, *p* = 0.395; GRP78, *p* = 0.509), despite 80.4% (74/92) patients displaying high GRP78 tumour expression and 81.5% (75/92) patients displaying high IGFBP‐2 tumour expression. However, larger published studies have linked IGFBP‐2 with prognosis in glioma, as a prognostic biomarker and therapeutic target both in tissue^14^, ^15^, ^33^, ^34^, ^35^, ^36^ and plasma.^20^, ^21^, ^37^, ^38^ In our study, tumour tissue with high IGFBP‐2 expression paired with low GRP78 expression were significantly associated with, a much shorter survival time (median = 4.0 months, *p* = 0.019). In other studies, higher GRP78 mRNA expression positively regulated overall and progression free survival in gastric cancer patients and was reported to be a positive marker for prognosis and chemotherapy response in breast cancer.^39^, ^40^ Moreover, drug induced GRP78 degradation in murine macrophages has been linked to prolonged ER stress and inflammation mediated by IL‐6 release, driving the tumorigenic microenvironment and cellular adaptation further exploiting adaptive mechanisms.^41^

The expression, pleiotropic functions and spatiotemporal dynamics of IGFBP‐2 and GRP78 appear to intricately shift in response to cellular context and adaptation. Redistribution of GRP78 via translocation to the surface of cells has been recorded in cancer cells in which a signal transducing role can be adopted.^42^, ^43^ The maintenance and radio sensitisation of mesenchymal GSCs have also been shown to be dependent upon cell surface GRP78 (csGRP78), adding to evidence that expression and function are context dependent.^25^ The mechanism of translocation is largely unknown but believed to involve additional binding partners as part of a context‐dependent process.^43^ There appears to be overlap between signalling pathways initiated by csGRP78 and IGFBP‐2. For example, csGRP78 binding to α‐2‐macroglobulin (α2M) is believed to stimulate invasion and metastasis.^44^ However, α2M is also believed to complex with IGFBP‐2 in the circulation preventing IGFBP‐2 proteolysis and modulating IGF signalling.^45^ Further crossover between the pathways have revealed that surface GRP78 can mediate IGF‐IR phosphorylation and activation of growth and survival pathways.^46^ Moreover, a feedback loop denotes that IGF‐1R mediated IGF‐I signalling can upregulate GRP78 via transduction through PI3K/AKT/mTORC1 signalling.^47^ IGF‐I has also been shown as a direct stimulator of PI3K/Akt and MAPK mediated expression and GRP78 cell surface redistribution in hepatoma cell lines.^46^ In addition, extracellular functions of IGFBP‐2 have been linked to the proliferative and invasive capacity of glioma cells via integrin β1‐mediated activation of ERK phosphorylation and nuclear translocation.^48^ Other downstream signalling cascades mediated by the integrin β1 involve ILK/NFκB signalling, FAK/ERK pathway activation and CD144 expression, as well as β‐catenin nuclear and cytoplasmic regulation.^49^, ^50^, ^51^, ^52^ Critically, the negative regulation between IGFBP‐2 and PTEN has been shown to be of clinical importance in triple negative breast cancer, glioblastoma and prostate cancer.^53^, ^54^, ^55^

Other IGFBPs have previously been identified as important malignancy biomarkers including IGFBP3; the most abundant IGFBP in the human serum. The ability of IGFBP‐3 to differentially affect cell survival and invasion, depending upon the extracellular environment, is influenced by the presence of GRP78 in human breast cancer cell lines.^23^ Mechanistic intricacies underlying the relationship between IGFBP‐2 and GRP78 remain to be elucidated. Future investigation will aim to determine the exogenous actions of IGFBP‐2 with and without the presence of GRP78, as well as the facilitation of IGFBP‐2 trafficking and oncogenic pathway activation via protein complexing, as suggested by other studies.^56^

## 4 CONCLUSION

Taken together, our experimental and clinical findings suggest that inverse levels of IGFBP‐2 and GRP78 may be adverse clinical prognostic markers in *IDH*‐wildtype glioblastoma. Further experimental studies into the mechanism of interaction and the potential for therapeutic manipulation are warranted.

## AUTHOR CONTRIBUTIONS

**Abigail J Harland:** Conceptualization (supporting); data curation (equal); formal analysis (equal); investigation (lead); methodology (equal); project administration (equal); validation (equal); visualization (lead); writing – original draft (lead); writing – review and editing (lead). **Claire M. Perks:** Conceptualization (equal); project administration (equal); resources (lead); supervision (equal); writing – review and editing (equal). **Paul White:** Data curation (equal); formal analysis (equal); investigation (equal); methodology (equal); resources (equal); software (equal); supervision (supporting); visualization (equal); writing – review and editing (equal). **Kathreena M Kurian:** Conceptualization (equal); formal analysis (equal); funding acquisition (lead); investigation (equal); methodology (equal); project administration (equal); resources (equal); supervision (equal); validation (equal); writing – review and editing (equal). **Hannah R Barber:** Conceptualization (lead); data curation (equal); formal analysis (equal); investigation (lead); methodology (equal); project administration (equal); validation (equal); writing – review and editing (equal).

## FUNDING INFORMATION

This work was supported by Cancer Research UK [grant number C18281/A29019]. Tissue samples were obtained from North Bristol NHS Trust as part of BRAIN UK, which has been supported by Brain Tumour Research, the British Neuropathological Society and the Medical Research Council.

## ETHICS STATEMENT

Tissue samples were obtained under BRAIN UK ethical approval. Study Ref: 15/001 (1). Brain UK REC Ref: 19/SC/0217 Renewal REC Ref: IRAS ID: 262890. Brain UK IRAS/ Ethics allows use of tissue with a waiver.

## Supporting information


**Figure S1.** Immunoblot technical repeats to analyse GRP78 and IGFBP‐2 protein expression in three independent passages of the control U3 NSC line and two GSC lines, *n* = 3. The order of sample loading was allocated at random to mitigate the effects of loading position on protein expression.
**Figure S2**. Figure 5. Immunoblots showing technical repeats of the co‐IP assay carried out on lysates extracted from G26 GSCs. IGFBP‐2 was pulled down using a GRP78 antibody in each case. The IgG2a antibody was used as an isotype control.

## Data Availability

The results in this paper are in part generated using Affymetrix Human Genome U133 Plus 2.0 Array data from Pollard et al. sourced from the NIH Gene Expression Omnibus (series GSE15209), https://www.ncbi.nlm.nih.gov/geo/query/acc.cgi?acc=GSE15209.
