# Supplementary material for: Insulin‐like growth factor binding protein‐2 and glucose‐regulated protein 78 kDa: Potential biomarkers affect prognosis in IDH ‐wildtype glioblastoma patients
Source: Cancer Med. 2023 May 22;12(13):14426–39. doi: 10.1002/cam4.6071 (PMC10358216; doi:10.1002/cam4.6071)
Supplement: Supplementary file 1 — Figure S1. Immunoblot technical repeats to analyse GRP78 and IGFBP‐2 protein expression in three independent passages of the control U3 NSC line and two GSC lines, n = 3. The order of sample loading was allocated at random to mitigate the effects of loading position on protein expression. Figure S2. Figure 5. Immunoblots showing technical repeats of the co‐IP assay carried out on lysates extracted from G26 GSCs. IGFBP‐2 was pulled down using a GRP78 antibody in each case. The IgG2a antibody was used as an isotype control. [file CAM4-12-14426-s001.docx]

Figure S1.


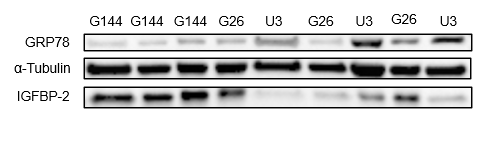


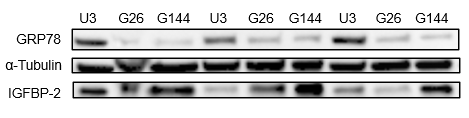


Figure S1. Immunoblot technical repeats to analyse GRP78 and IGFBP-2 protein expression in three independent passages of the control U3 NSC line and two GSC lines, n=3. The order of sample loading was allocated at random to mitigate the effects of loading position on protein expression.

Figure S2.


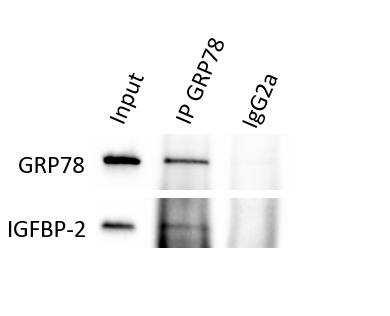

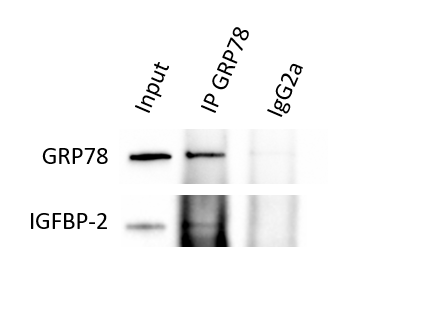


Figure S2. Figure 5. Immunoblots showing technical repeats of the co-IP assay carried out on lysates extracted from G26 GSCs. IGFBP-2 was pulled down using a GRP78 antibody in each case. The IgG2a antibody was used as an isotype control.
